# Evaluating pharmacists’ knowledge, attitude, and practices toward amiodarone cross-reactivity with iodine: a cross-sectional pilot study

**DOI:** 10.7717/peerj.13665

**Published:** 2022-07-08

**Authors:** Nura Abdullah Alshehab, Munirah Abdullah Alhumaid, Mohammed Abdulrazaq Alabdulwahed, Abdulaziz Saleh Almulhim

**Affiliations:** Department of Pharmacy Practice, College of Clinical Pharmacy, King Faisal University, Al Ahsa, Saudi Arabia

**Keywords:** Amiodarone, Iodine allergy, Shellfish allergy, Radiocontrast media allergy, Hypersensitivity, Pharmacist

## Abstract

**Introduction:**

Iodine is a vital mineral of the human body that acts by maintaining the health of the thyroid gland. Research has shown that iodine-rich food allergy (*i.e*., seafood allergy) is not caused by iodine itself; instead, it is caused by other proteins including tropomyosin and parvalbumin in shellfish and fish, respectively. Amiodarone is a commonly used antiarrhythmic agent containing a significant amount of iodine.

**Objective:**

This study’s objective was to assess pharmacists’ knowledge, attitude, and practices toward the misconception of iodine allergy and the cross-reactivity with amiodarone.

**Methods:**

In February 2020, a cross-sectional study was conducted by sending out an online survey to three pharmaceutical organizations (Saudi Pharmaceutical Society, Kuwait Pharmaceutical Association, and Oman Pharmaceutical Society). Additionally, an electronic questionnaire was administered to pharmacists attending the Dubai International Pharmaceuticals and Technologies Conference and Exhibition 2020 (DUPHAT). Chi-square or Fisher’s exact test, when appropriate, were used to compare categorical variables. The statistical analyses were carried out using SPSS software.

**Results:**

Data were collected from 66 respondents. However, only 61 (92.4%) were included in the final analysis following the exclusion of incomplete responses. The mean age of participants was 35 ± 8.48 years. The majority of participants did not have the Board of Pharmacy Specialties Certification (54.1%). Moreover, (41%) of participants licensed as pharmacists with more than 10 years of initial pharmacy licensure. Forty-three (70.5%) of participants had the misconception that iodine allergy should be considered before amiodarone administration, 20/32 (62.5%) of whom considered iodine allergy alone thought that premedication with corticosteroids and/or antihistamines is necessary. Concerning iodine allergy and amiodarone use, there was no significant difference in knowledge between the pharmacists who have board certification and those who did not.

**Conclusion:**

Pharmacists’ misconception concerning iodine allergy and cross-reactivity with amiodarone was evident. Implementation of educational programs targeting pharmacists is necessary to correct these misconceptions.

## Introduction

Iodine is an essential micronutrient needed for the synthesis of thyroid hormones which regulate many physiological processes (FAO/WHO, 2005; Schabelman & Witting, 2010). It is ubiquitous in the western diet, and its concentration varies depending on the type of diet (FAO/WHO, 2005). Iodine’s concentration in seafood and shellfish is high (FAO/WHO, 2005; Nerhus et al., 2018); hence seafood and shellfish allergy were previously thought to be due to iodine (Shehadi, 1975). It is currently well accepted that an allergy to shellfish and seafood is mediated by immunoglobulin (Ig)-E and is caused by protein tropomyosin (TM) (Schabelman & Witting, 2010). Iodine, since it is necessary for the synthesis of thyroid hormones, cannot cause an allergy (Schabelman & Witting, 2010). Amiodarone is a broad-spectrum antiarrhythmic agent that is used for supraventricular and ventricular arrhythmias (Narayana, Woods & Boos, 2011). Amiodarone contains 37% iodine by weight, and is close in structure to thyroid hormones (Narayana, Woods & Boos, 2011). Like amiodarone, iodinated contrast media contains high concentration of iodine. Several studies were conducted to evaluate physicians’ misconceptions regarding iodine allergy and cross-reactivity with iodinated radiocontrast agents (Sampson et al., 2019; Beaty, Lieberman & Slavin, 2008; Baig et al., 2014). The misconception was common among cardiologists, radiologists, and emergency physicians (Sampson et al., 2019; Beaty, Lieberman & Slavin, 2008; Baig et al., 2014). The allergic reactions associated with iodinated contrast media are usually non-immunoglobulin (Ig)-E mediated, and are believed to be mediated by histamine release and complement activation (Singh & Daftary, 2008; Lieberman & Seigle, 1999). This histamine release is independent of the iodine content of the iodinated radiocontrast media (Lieberman & Seigle, 1999). The origin of this misconception is believed to be originated by Shehadi (1975). Little is known about pharmacists’ misconceptions regarding iodine allergy and amiodarone cross-reactivity. This study’s objective was to determine pharmacists’ knowledge, attitude, and practices about the misconception of iodine allergy and the cross-reactivity with amiodarone.

## Methods

### Design, inclusion criteria, and study sample

This was a cross-sectional study. Data were collected as previously described in Almulhim, Al-Dahneen & Alsowaida (2020). In short, an electronic link was sent to three scientific organizations: Saudi Pharmaceutical Society (SPS), Kuwait Pharmaceutical Association (KuPhA), and Oman Pharmaceutical Society (OmPhS). Additionally, a purposive sampling technique was conducted (Rothman, Gallacher & Hatch, 2013; Babar, 2020). Pharmacists attending the Dubai International Pharmaceuticals & Technologies Conference & Exhibition 2020 (DUPHAT) were approached and invited to participate in this study by a pharmacy intern (N.A) who was trained and prepared to collect data. Participants were provided with a tablet device and self-administered the survey. Only licensed pharmacists were allowed to participate. SurveyMonkey® is an online questionnaire generator that was utilized to design and distribute the questionnaire. The survey was designed to allow only one attempt per respondent. The survey was available between February 27^th^ and March 31^st^, 2020.

### Contents of the tool

The survey was not derived from any previously published questionnaire. However, the flow and the concept of the items were similar to that of Beaty, Lieberman & Slavin (2008). The questionnaire consisted of four domains. The first domain was a cover letter that provided general information about the purpose of the survey. To proceed to the questionnaire, the participant had to agree on the terms and his/her willingness to participate (Table 1). The second domain consisted of demographic data (age, gender, year of initial pharmacy licensure, pharmacy board certification status, and finally country where participant work). The third domain was to assess the respondents’ general knowledge about amiodarone and as a distractor to obscure the primary purpose of the study. The last question in the third domain was select all that apply; a branch logic was created to customize the path of the survey. To assess their practices, respondents who selected shellfish or iodine allergy were directed to the fourth domain where they had to answer whether premedication with corticosteroids or antihistamine would be needed or not before initiation of amiodarone for both types of allergies, respectively. In case other options were selected, respondents were directed to the last question in the fourth domain. The last question was a distractor to assess pharmacists’ attitude toward iodine allergy documentation in patients’ charts.

**Table 1:** Survey items. Survey items used to evaluate pharmacists’ knowledge, attitude, and practices toward amiodarone cross-reactivity with iodine.

First domain (Cover letter)
You are being asked to participate in a research study. Your participation in this research study is voluntary and you do not have to participate. This document contains important information about this study and what to expect if you decide to participate. The purpose of this study is to assess pharmacists’ knowledge about amiodarone. Your participation in this online survey will help the scientific and clinical communities better understand knowledge gaps and needs for education. We estimate that it will take a maximum of 3 min of your time to complete the questionnaire. Risks to participants are considered minimal. There will be no costs for participating, nor will you benefit directly from participating. The information that you give will be anonymous. We have taken all reasonable measures to protect your identity and responses. The questions in this survey do not ask you to reveal any personal identifying information such as your name, the data are encrypted and stored in a password-protected database, and IP addresses are not collected. This questionnaire is intended for licensed pharmacists only. Completing the survey will indicate your willingness to participate in the survey and consent for your responses to be used for research.
Second domain
1. What is your age?
2. Gender: a. Male b. Female c. Prefer not to answer
3. Year of initial pharmacy license: a. Less than 5 years b. 5–10 years c. More than 10 years
4. Board of Pharmacy Specialties Certification: a. Yes b. No
5. In what country do you work a. Saudi Arabia b. United Arab of Emirates c. Oman d. Kuwait e. Bahrain f. Other countries
Third domain^a^
1. Amiodarone has iodine in its structure: a. True* b. False
2. Which of the following can be caused by amiodarone? Check all that apply: a. Liver disorders* b. Thyroid disorders* c. Lung disorders*
3. Which of the following warrants dose adjustment prior to amiodarone initiation? a. Digoxin b. Warfarin c. Both digoxin and warfarin*
4. Which of following allergies should be considered prior to amiodarone use? Check all that apply^b^: a. Sulfa allergy* b. Peanut allergy* c. Animal dander* d. Shellfish allergy* e. Iodine allergy f. None of the above
Fourth domain
1. In a patient with a known iodine allergy, do you recommend premedication with corticosteroids and/or antihistamines prior to amiodarone initiation? a. Yes b. No*
2. In a patient with a known shellfish allergy, do you recommend premedication with corticosteroids and/or antihistamines prior to amiodarone initiation? a. Yes b. No*
3. Which of the following allergies do you think should be in your pharmacy computer system and/or electronic medical records? Check all that apply: a. Sulfa allergy* b. Peanut allergy* c. Animal dander* d. Shellfish allergy* e. Iodine allergy f. None of the above

**Notes:**

a The purpose of this domain was to act as a distractor.

b A branch logic was activated; those who chose iodine or shellfish allergy were directed to the fourth domain, while others were directed to the last question of the fourth domain.

* Correct answers.

### Content validity

The instrument was face validated qualitatively by an expert panel consisting of pharmacy fellows (*n* = 5), and a pharmacy professor (*n* = 1). Neither were members of the aforementioned professional societies. During the face validation phase, respondents found that questions appropriate and interpretable. Modifications to the tool were made based on the recommendations and suggestions pointed by the panel.

### Ethical approval

The initial Institutional Review Board (IRB) was obtained from The University of Arizona. The study met the exemption criteria (protocol number: 2019-007, July 16th, 2019). Due to travel circumstances and inability to conduct study at the country where first IRB approval was obtained, and to account for practices variation, different traditions, laws, and mores from place to place. The authors obtained another IRB from King Faisal University as the study was carried out in the Middle East (Ethical Application Ref: KFU-REC-2022-MAY-EA000615). No personal information was asked in the survey (*i.e*., name or contact number). Survey settings were modified to disable location identification prior to distribution. Additionally, written consent (*i.e*., in the form of a cover letter) was also provided to explain the risks, willingness to participate, and study purpose Table 1. Respondents had to agree and give their consent to proceed to the survey.

### Statistical analysis

Analyses were carried out using Statistical Package for the Social Sciences (SPSS) software for Windows (version 23.0). Descriptive statistics were summarized as percentages (*i.e*., frequencies) and means (± standard deviation [SD]). Chi-square or Fisher’s exact test, when appropriate, were used to compare categorical variables. All *p*-values less than 0.05 were considered statistically significant.

## Results

### Demographic characteristics of participants

**Figure 1:**
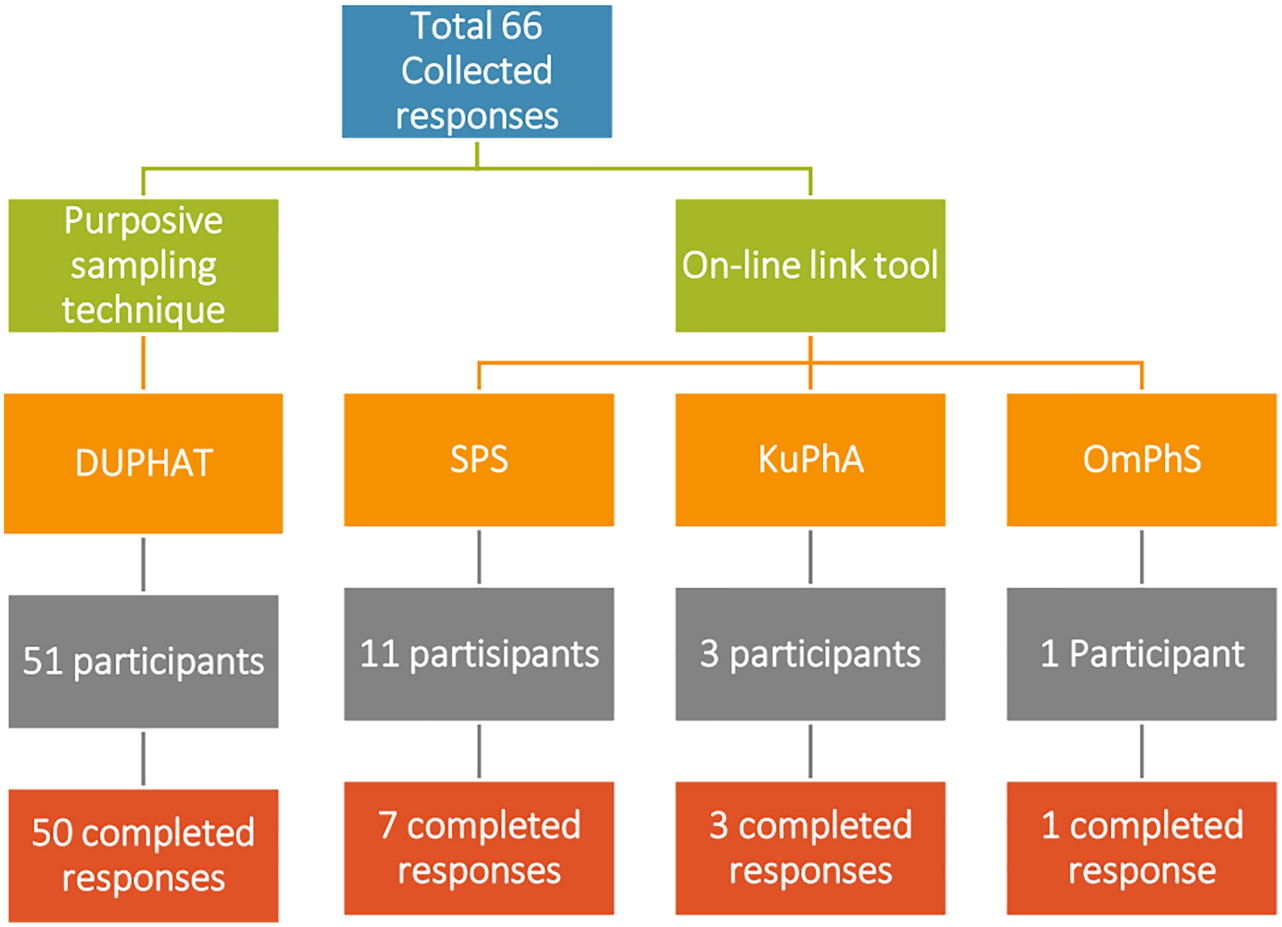
Sample collection with number of completed responses.

Sixty-six responses were collected from licensed pharmacists; however, only 61 were completed with a completion rate of 92% (Fig. 1). Only 11/61 (18%) responses received from the professional organizations in the countries of the Gulf Region using the online link. SPS contributed to 7/61 (11.5%) completed responses, compared to KuPhA 3/61 (4.9%) responses, and OmPhS 1/61 (1.6%) response. The majority of respondents were females 39/61 (63.9%) as shown in Table 2. Most of the respondents work in Saudi Arabia 29/61 (47.5%) with most of them being in Riyadh 11/61 (18%). The mean age of respondents was 35 ± 8.48 years. Most of the respondents did not have a Board of Pharmacy Specialties Certification 33/61 (54.1%) but most of them had more than 10 years of initial pharmacy licensure 25/61 (41%).

**Table 2:** Demographic characteristics of participants.

Characteristic	(N = 61)
Age (Mean ± SD)	35 ± 8.48
Gender—no. (%)	
Male	22 (36.1)
Female	39 (63.9)
Prefer not to answer	0
Year of initial pharmacy license*—no. (%)	
Less than 5 years	19 (31.1)
5–10 years	17 (27.9)
More than 10 years	25 (41)
Board of Pharmacy Specialties Certification—no. (%)	
Yes	28 (45.9)
No	33 (54.1)
Country—no. (%)	
Saudi Arabia	29 (47.5)
United Arab of Emirates	24 (39.3)
Oman	1 (1.6)
Kuwait	4 (6.6)
Bahrain	1 (1.6)
Other countries	2 (3.3)
Responses from professional organizations in the Gulf Region—no. (%)	
SPS	7 (11.5)
KuPhA	3 (4.9)
OmPhS	1 (1.6)

**Notes:**

* Years of licensed pharmacy practice experience.

SPS: Saudi Pharmaceutical Society. KuPhA: Kuwait Pharmaceutical Association. OmPhS: Oman Pharmaceutical Society.

### Pharmacists’ knowledge about amiodarone

Pharmacists were first asked general information about amiodarone, only 37/61 (60.7%) of participants knew that amiodarone has iodine in its structure. Then, they were asked about the disorders that can be caused by amiodarone. Responses obtained include thyroid diseases 30 (49.2%) lung diseases 18/61 (29.5%) and liver diseases 29/61 (47.5%). Forty-seven 47/61 (77%) of participated pharmacists believed that both digoxin and warfarin dosage should be adjusted before amiodarone initiation.

### Pharmacists’ attitude, and practices toward iodine allergy and cross-reactivity with amiodarone

**Figure 2:**
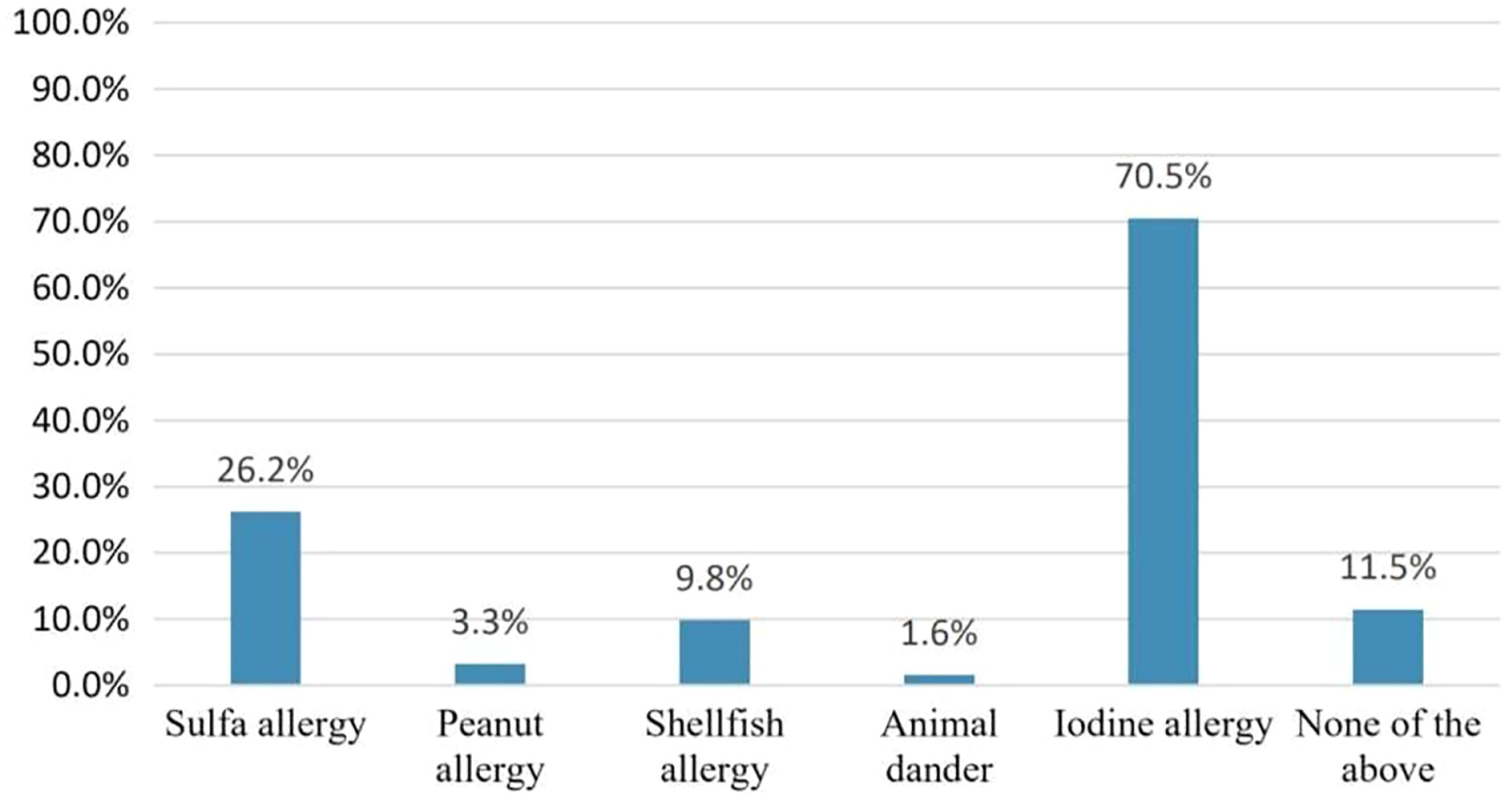
The following allergies should be considered prior to amiodarone use.

**Figure 3:**
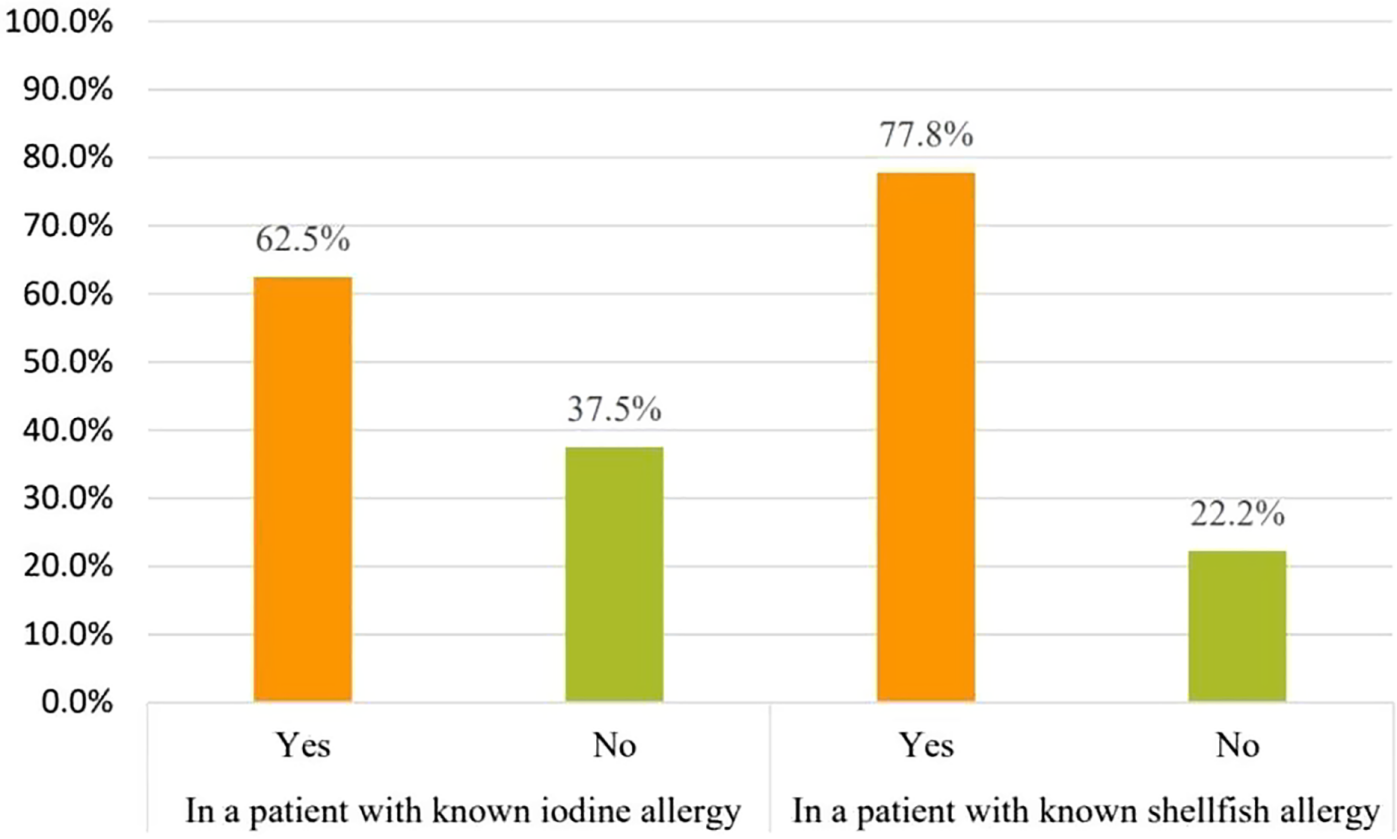
Do you recommend premedication with corticosteroids and/or antihistamines prior to amiodarone initiation in these patients with known allergies?

**Figure 4:**
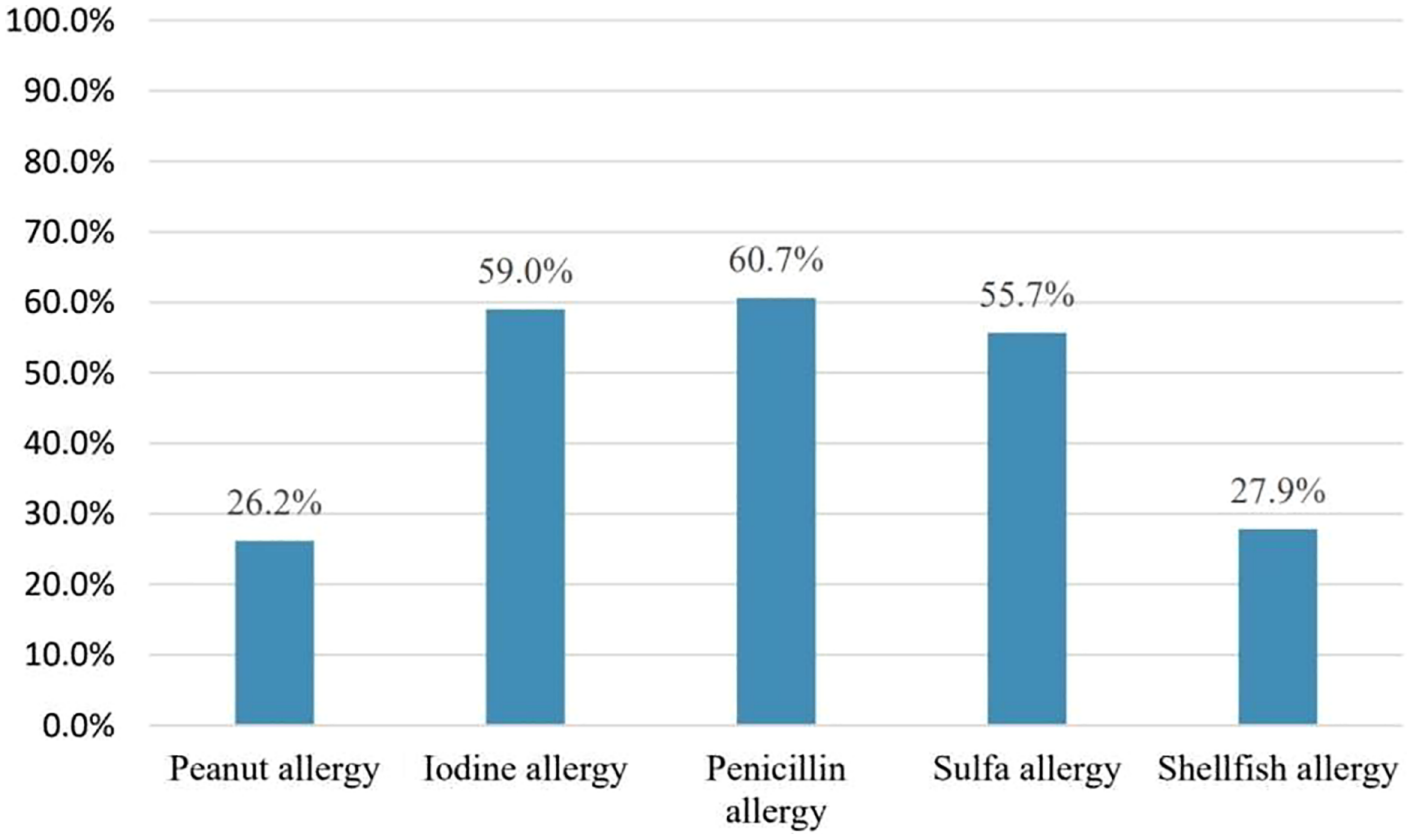
Which of the following allergies do you think should be in your pharmacy computer system and/or electronic medical record?

Pharmacists were asked which type of allergy should be considered before amiodarone use to assess the misconception that amiodarone cross-reactivity could be caused by iodine allergy. Forty-three pharmacists 43/61(70.5%) responded that iodine allergy should be considered. However, only six pharmacists 6/61 (9.8%) believed that shellfish allergy should be considered Fig. 2. On the other hand, 32/43 (74.4%) considered the need to confirm the presence of iodine allergy alone before amiodarone use. Concerning iodine allergy and premedication, 20/32 (62.5%) pharmacists believed that premedication with corticosteroids and/or antihistamines in case of iodine allergy alone is necessary. On the other hand, out of 36 pharmacists who chose to consider shellfish allergy before amiodarone use, 28/36 (77.8%) agreed that premedication should be given in case shellfish allergy Fig. 3. Concerning allergy documentation, 36/61 (59%), 17/61 (27.9%), 37/61 (60.7%), and, 34/61 (55.7%) of the respondents thought that iodine allergy documentation in addition to shellfish, penicillin, and sulfa allergy respectively should be included in patients’ medical records or the pharmacy computer system Fig. 4.

### Association of need for premedication in patients with known iodine or shellfish allergy and board of pharmacy specialties certification

The ownership of a Board of Pharmacy Specialties Certification did not significantly impact the decision making of the necessity for premedication with corticosteroids and/or antihistamines for iodine or shellfish allergy, *X*^*2*^ (1, *N* = 61) = 0.209, *p* = 0.647, *X*^*2*^ (1, *N* = 61) = 0.209, *p* = 0.434, respectively.

### Knowledge difference

**Figure 5:**
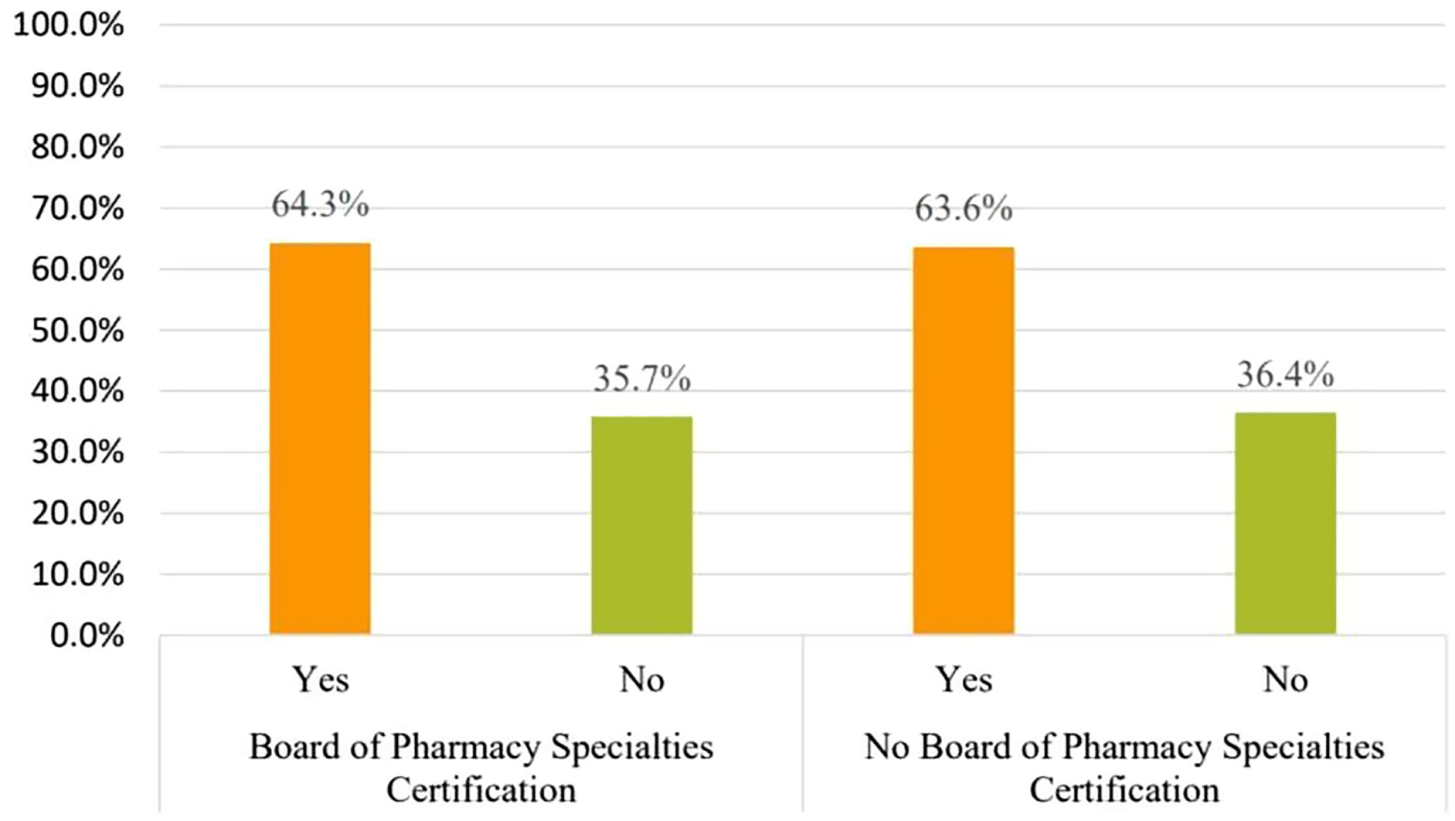
The influence of Board of Pharmacy Specialties Certification on pharmacists’ perspective of considering iodine allergy prior to amiodarone use.

In comparison between pharmacists who have a Board of Pharmacy Specialties Certification 28/61 (45.9%) and those who do not have a Board of Pharmacy Specialties Certification 33/61 (54.1%), having the certification had no significant difference on considering iodine allergy before amiodarone use, *X*^*2*^ (1, *N* = 61) = 0.506, *p* = 0.477 (Fig. 5).

## Discussion

This cross-sectional study was performed to determine the pharmacists’ knowledge, attitude, and practices toward iodine allergy misconception and the cross-reactivity with amiodarone. Although general knowledge about amiodarone was modest, our findings suggest that iodine allergy and amiodarone cross-reactivity misconception among pharmacists was common. This misconception was evident when respondents were asked about the iodine allergy to be considered before amiodarone use or administration 43/61 (70.5%). Additionally, this misbelief was apparent when respondents who chose iodine allergy alone 32/43 (74%) asked about the need for premedication with corticosteroids and/or antihistamines 20/32 (62.5%). This misconception was not affected by the status of board certification.

Although amiodarone cross-reactivity in patients with documented iodine allergy was reported in several case reports and case series, other factors could have contributed to the anaphylactoid reactions reported in these studies in which patients developed anaphylaxis, angioedema, urticaria, dyspnea and bronchoconstriction (Stafford, 2007; Serhan Özcan et al., 2014). On the other hand, Snider, Boyd & Carnes (2008) reported a case of a patient with a history of anaphylaxis reaction to iodine documented with two previous reactions to iodinated contrast dye, who has been taking amiodarone 200 mg orally once daily and tolerated it throughout the 6-month follow-up period without any allergic reactions. Although the patient was taking diphenhydramine concurrently, the possibility of diphenhydramine in masking a hypersensitivity reaction to amiodarone was ruled out based on the relatively low dose the patient was receiving and the lack of symptoms after decreasing the diphenhydramine dose to 25 mg per week (Snider, Boyd & Carnes, 2008). Another case confirmed that a shellfish allergy does not necessarily imply an iodine allergy was reported by Beall and colleagues in which a patient with a history to shellfish allergy has been receiving an oral amiodarone chronically without an developing any allergic reaction (Beall, Mahan & Blau, 2007). likewise, Brouse and colleagues reported a case series of three patients with listed iodine allergy who were administered amiodarone with no reactions observed (Brouse & Phillips, 2005). Similarly, a retrospective study conducted at two academic medical centers found that only one patient out of 234 (0.4%) with listed iodine allergy developed allergy following amiodarone exposure. Authors concluded that the use of amiodarone in this population is justifiable if clinically indicated (Lakshmanadoss et al., 2012).

The results of our study are in concordance with previously reported studies (Sampson et al., 2019; Beaty, Lieberman & Slavin, 2008; Baig et al., 2014). Among cardiologists and radiologists, 50% and 37%, respectively, responded that they would withhold or recommend premedication before administering iodinated radiocontrast agents in patients with documented seafood allergy (Beaty, Lieberman & Slavin, 2008). Similarly, another study was conducted in the United Kingdom to determine cardiologists’ practice in terms of shellfish or iodine allergy. Fifty-six percent would consider pretreatment (Baig et al., 2014). More recently, a study was conducted at the emergency department level in more than three practice sites found that among emergency physicians and radiologists, 23% and 13% would premedicate before administering iodinated contrast media, respectively (Sampson et al., 2019).

To determine the impact of education on iodine allergy and iodinated contrast media misbelief, Westerman-Clark et al. (2015) conducted a pre-post study in which knowledge of participants, including physicians of different specialties, was evaluated based on their answers to a set of questions before and after an education session. The mean correct responses were higher in the post-test, which was statistically significant compared to the pre-test scores.

Although most of the participants in this study were aware of the structure of amiodarone, our findings can create a common ground that lacking baseline knowledge among healthcare professionals can mislead their decision making in regards to medication of choice. Therefore, we need to increase the awareness of amiodarone related allergies and its irrelevance to iodine to all healthcare professionals, including but not limited to pharmacists.

The current study has several limitations. First, the number of participants was small; this was mainly due to the low response rate the authors encountered with the online survey. Moreover, it was not easy to find licensed pharmacists at DUPHAT conference, as it is open to physicians, students, and workers from companies and not only to pharmacists. The majority of the participants were from one city in Saudi Arabia and from the United Arab of Emirates, which limits the generalizability of our results to other countries in the Gulf region. Second, formal sample size calculation was not conducted during the planning of this study, as none of the above-mentioned scientific organizations were able to provide the authors with a specific number of registered licensed pharmacists. Their email listservs were not powered to categorize their members based on licensure (*i.e*., pharmacists *vs*. pharmacy technicians *vs*. pharmacy students). We suggest more extensive studies to be conducted, which can be more revealing and yield useful insight into this matter.

## Conclusion

Pharmacists’ misconception concerning iodine allergy and cross-reactivity with amiodarone was evident. The term iodine allergy should go obsolete as such term can cause confusion and may affect pharmacists’ decision making. Developing educational programs are essential to help to correct these misconceptions.

### Authorship

All named authors meet the International Committee of Medical Journal Editors (ICMJE) criteria for authorship for this article, take responsibility for the integrity of the work as a whole, and have given their approval for this version to be published.

## Supplemental Information

10.7717/peerj.13665/supp-1 Supplemental Information 1 Raw Data of Pharmacist Knowledge, Attitude, and Practices toward Amiodarone.

## Additional Information and Declarations

### Data Availability

The following information was supplied regarding data availability: The raw data are available in the Supplemental File.

We thank the participants of the study.
